# Fifteen-Year Follow-Up of a Case of Surgical Retreatment of a Single Gingival Recession

**DOI:** 10.1155/2018/3735162

**Published:** 2018-01-27

**Authors:** Luca Francetti, Silvio Taschieri, Nicolò Cavalli, Stefano Corbella

**Affiliations:** ^1^Department of Biomedical, Surgical and Dental Sciences, Università degli Studi di Milano, Milan, Italy; ^2^IRCCS Istituto Ortopedico Galeazzi, Milan, Italy

## Abstract

**Purpose:**

The aim of the present case report was to describe the retreatment of the single gingival recession in aesthetic area, in the presence of scar formation and consequent impairment of aesthetic appearance.

**Methods:**

A young patient with one single recession of 4 mm of 2.1 was treated with coronally advanced flap and subepithelial connective tissue graft, through a microsurgical approach that aimed at the removal of the scarred fibrous tissue. The intervention was performed using a surgical microscope as a magnification device.

**Results:**

Fifteen years after the surgical treatment, a substantial stable resolution of the gingival recession could be observed. Moreover, a further improvement of the aesthetic appearance could be observed.

**Conclusions:**

This case report suggests that periodontal microsurgery could be an effective approach for the retreatment of gingival recessions and, in long-term evaluation, to reduce the aesthetic problem due to the presence of scar formation. Further studies with a larger sample size are needed to better evaluate its efficacy.

## 1. Introduction

Gingival recession is the result of the apical migration of the gingival margin, thus exposing portions of the tooth root that can cause aesthetic impairment when it occurs in anterior regions of the mouth [1, 2]. Moreover, gingival recessions can predispose to root caries development and/or dentinal hypersensitivity [1, 3].

Even though a large debate exists on the etiology of gingival recession, many factors were related to the initiation and development of the apical migration of the gingival margin [4, 5]. In particular, oral piercings, orthodontic forces and appliances, peculiar anatomical conditions in presence of particularly predisposed gingival biotype (thin and scalloped one in particular), and inadequate oral hygiene maneuvers could be related to the presence of recessions [6–10].

Since in most cases gingival recessions remain totally unperceived by patients and asymptomatic, thus limiting the indications to treatment [11], in some cases they could be related to a number of conditions that can cause discomfort to patients or they can be misinterpreted as a sign of periodontitis. Indeed, gingival recessions were recognized as one factor related to dentinal hypersensitivity [3, 12], the formation of carious or noncarious cervical lesions, difficulties in maintaining oral hygiene, and aesthetic impairment [13].

Several surgical techniques were proposed and described in scientific literature for the treatment of gingival recessions [14]. Coronally advanced flap (CAF) with or without connective tissue graft (CTG) is one of the most common surgical techniques in this field, and its application was widely validated by several randomized controlled clinical trials [15–17] and systematic reviews of the literature [18, 19].

One recent review of the literature [14] reported, for cases treated with CAF, only a mean root coverage ranging from 34.0% to 86.7% and a percentage of recessions that resulted complete coverage ranging from 11.0% to 60.0%. As for CAF + CTG, the proportion of recession with complete root coverage ranged from 18.1% to 97.0%, while the mean root coverage ranged from 64.4% to 96.0%.

Since in most cases even partial coverage of the gingival recession could lead to a reduction of symptoms related to dentinal hypersensitivity, an inappropriate technique, in particular regarding the flap management, was related to scar formation and, consequently, an impairment of the aesthetic aspect [14].

The aim of the present paper was to describe the long-term outcomes of a retreatment of one single recession in aesthetic area, treated with a microsurgical approach with the aid of a surgical microscope.

## 2. Case Presentation

A 21-year-old female patient presented in 2002 with vague symptoms of dentinal hypersensitivity and with a significant dissatisfaction with the aesthetics due to the gingival recession of the tooth 2.1 (left maxillary central incisor) and for the appearance of the surrounding gingival tissues. The patient reported that in 2000 two surgical interventions were already performed to treat the gingival recession after orthodontic treatment and they both resulted in incomplete root coverage and in the formation of scars in the site of surgery which importantly compromised the aesthetics. The exact surgical procedure undergone by the patient was not known. Medical anamnesis was collected before surgery, and the patient was classified as ASA-1 (following the American Academy of Anaesthesiologists classification), having no relative or absolute contraindications to surgical treatment. The subject did not smoke at the time of intervention.

**Figure 1:**
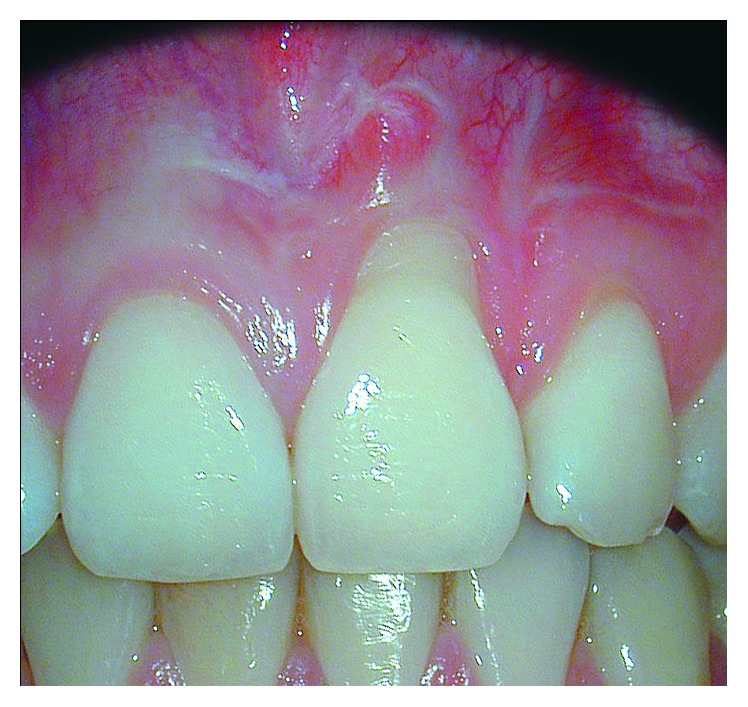
Clinical conditions at baseline. It was noticeable the scar tissue in the region of the intervention and the persistence of a gingival recession of 2.1.

The clinical examination revealed the presence of a Miller I [20] recession of 4 mm on 2.1 and of 1 mm on 2.2. No periodontal pockets deeper than 3.5 mm were found, confirming the absence of active periodontitis. In the region of 2.1, a small band of keratinized tissue (1 mm) was found apical to the gingival margin, and scars extending from the region of 1.1 to 2.2 were found (Figure 1). All teeth were vital and stable, the full-mouth bleeding score was 15%, and the full-mouth plaque score was 15%. From the aesthetic point of view, we evaluated a pink esthetic score (PES) of 4 (on a scale of 10) [21] and a white esthetic score (WES) of 6 (on a scale of 10) [22].

The patient was informed about the clinical conditions and about the treatment alternatives that were, in this particular case, limited. The first proposed option was to perform a surgical retreatment of the gingival recession, aiming to remove the scars through the surgical approach; as an alternative in order to treat the dentinal hypersensitivity, a topical desensitizing treatment was proposed, in case the patient preferred to avoid a surgical approach. After complete explanation of the procedures that could be adopted, the patient decided to undergo a surgical retreatment and signed an inform consent form, approving also the publication of clinical pictures.

### 2.1. Surgical Procedure

The surgical procedure was performed in 2003 by one oral surgeon with more than ten years of experience in periodontal plastic surgery and a specific training for the use of surgical microscope (LF). The surgery was performed under magnification obtained by a surgical microscope (Universa 300, MÖLLER-WEDEL GmbH & Co. KG, Wedel, Germany).

**Figure 2:**
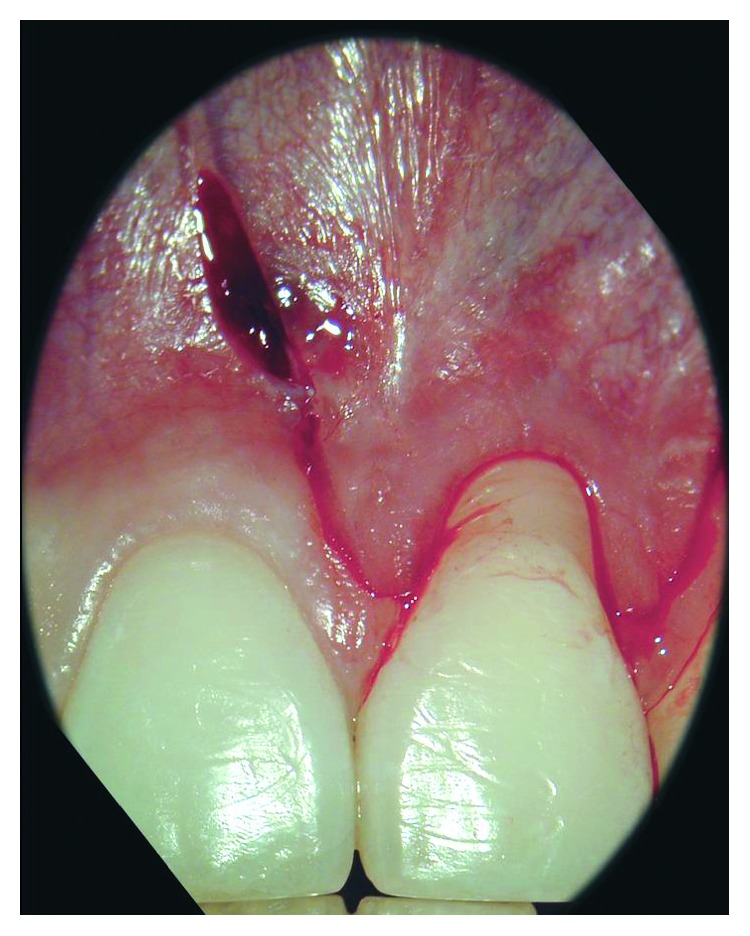
Trapezoidal flap was prepared. The vertical incisions extended significantly beyond the mucogingival junction and were beveled.

**Figure 3:**
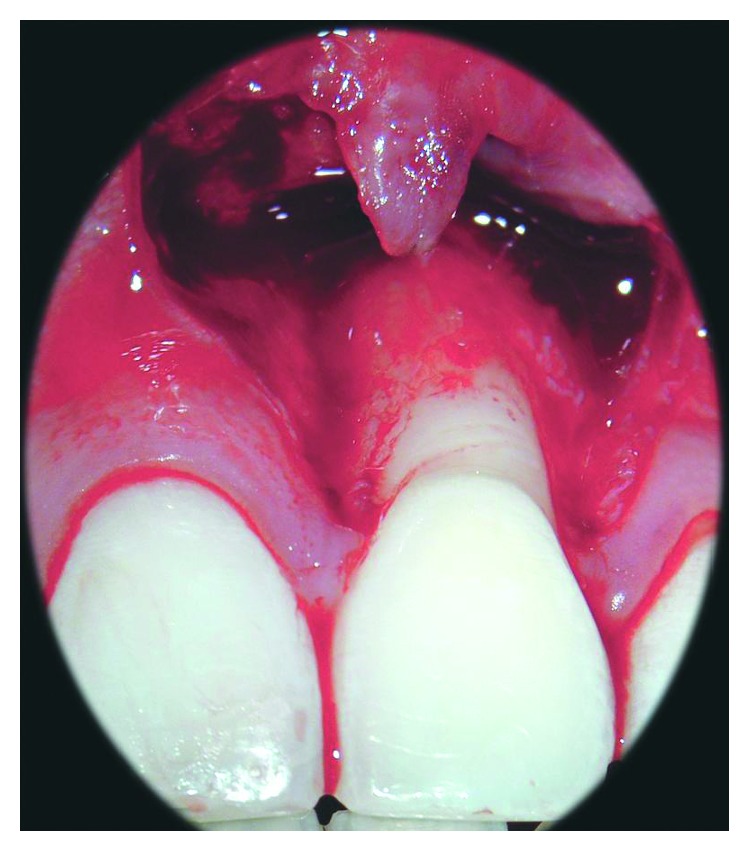
After the dissection of the scar tissues that limited the mobility of the flap, it can be repositioned coronally without tensions.

Local anesthesia with articaine 4% + epinephrine 1 : 100,000. was performed buccally in the site of the intervention A trapezoidal flap, made of two beveled and slightly divergent vertical incisions extending beyond the mucogingival junction, was elevated using microsurgical blades. The vertical incisions were connected by one sulcular, which was performed in the gingival sulcus of the affected tooth (Figure 2). A split-thickness flap was carefully elevated, extending beyond the mucogingival junction, leaving the periosteum attached to the bone surface and untouched. In the region of the mesial and distal papilla of the treated tooth, the epithelium was removed, leaving the vascular connective tissue in site. The exposed root surface was accurately debrided through sharp curette (Figure 3).

**Figure 4:**
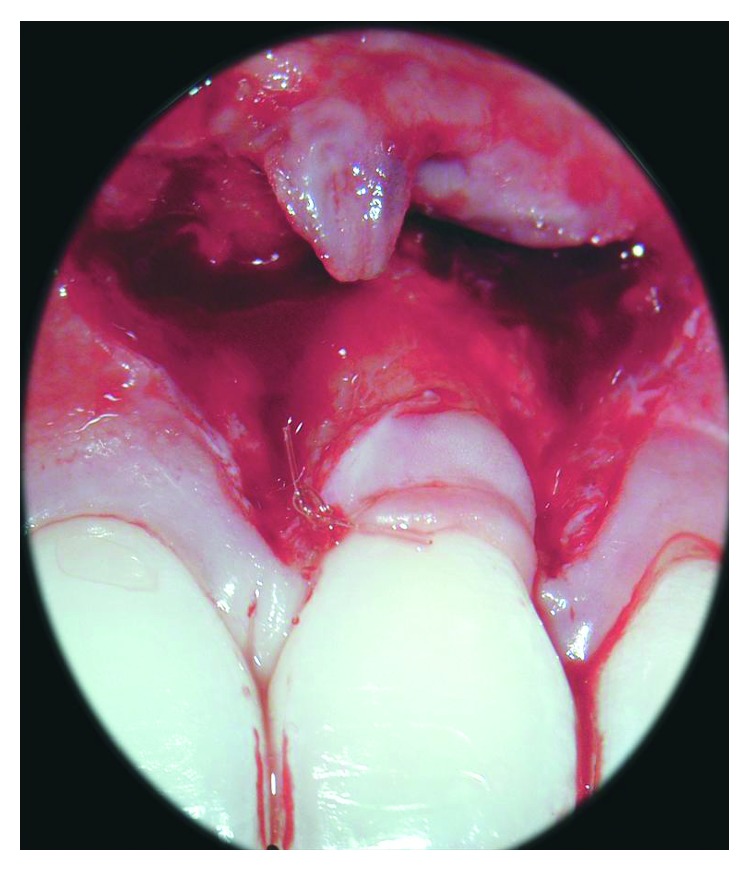
A connective tissue graft was placed in the site of the recession and stabilized by the use of resorbable sutures.

Afterwards, a connective tissue graft (1-2 mm thick) was harvested from the palate in the region extending from the second premolar to the second molar using the trap door approach [23] and trimmed as necessary to remove visible epithelium. The graft was then placed to cover the recession defect, at the level of the CEJ, and stabilized using resorbable sutures (Monocryl® 5-0, Ethicon, Inc., Johnson & Johnson, Piscataway, NJ, USA) anchored to the periosteum (Figure 4).

**Figure 5:**
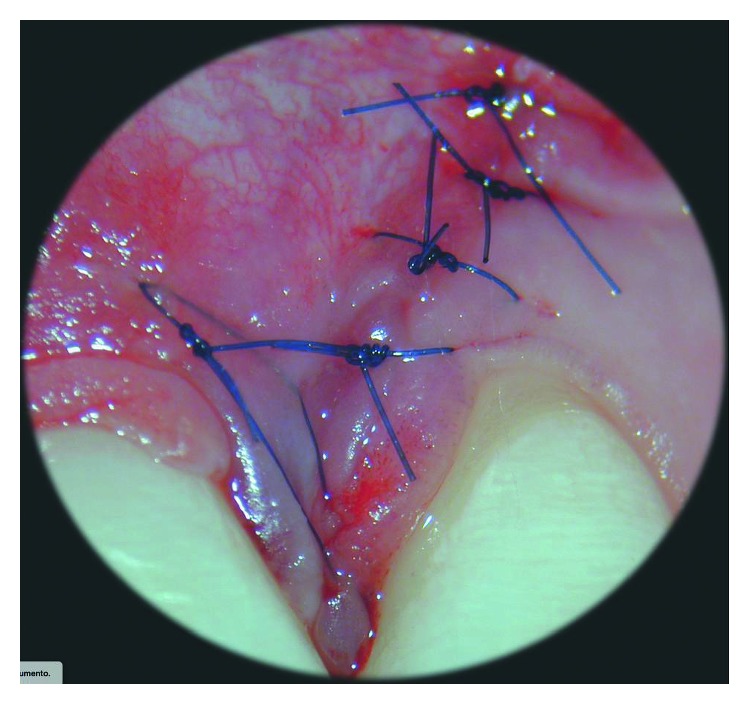
Detail of the sutures placed for vertical incisions.

**Figure 6:**
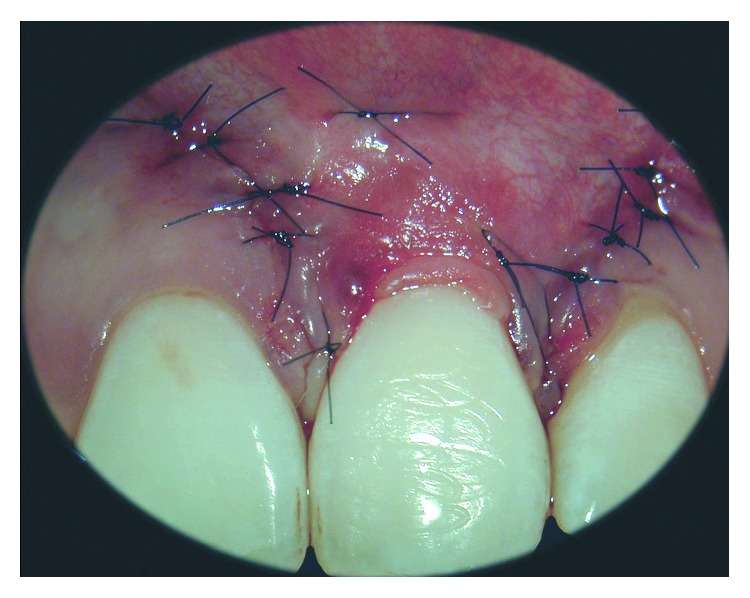
The flap was sutured in a more coronal position, and the connective tissue graft was partially covered.

In order to obtain a coronal repositioning of the flap, excluding tensions deriving from muscles, the elevated flap was released through partial-thickness incisions of muscular insertions to the periosteum deep apically. Then, the flap was sutured with interrupted sutures on the vertical release incisions (Deknalon® 6-0 Deknatel, Genzyme GmbH, Lübeck, Germany) and one sling suture (Deknalon 6-0 Deknatel, Genzyme GmbH, Lübeck, Germany) (Figures 5 and 6).

The patient was advised to avoid any trauma in the region of surgery and not to consume hard food during the first three days. Ketoprofen and lysine salt 80 mg was prescribed twice a day for two days for inflammation and pain control. Toothbrushing in the region of surgery was avoided for three weeks, and plaque control was obtained using 0.5% chlorhexidine digluconate spray, applied twice a day. After this period, the patient was instructed to resume toothbrushing using ultrasoft bristles for three more weeks. Subsequently, standard oral hygiene procedures were reintroduced.

**Figure 7:**
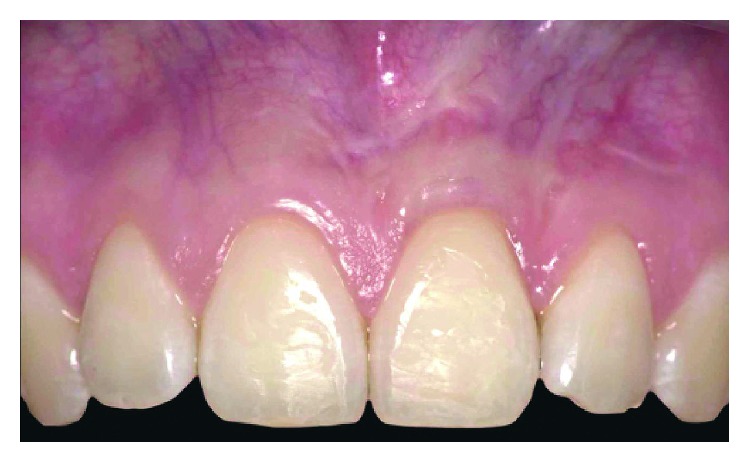
Clinical conditions one year after surgical intervention. If compared to baseline, a significant reduction of gingival recession could be observed.

**Figure 8:**
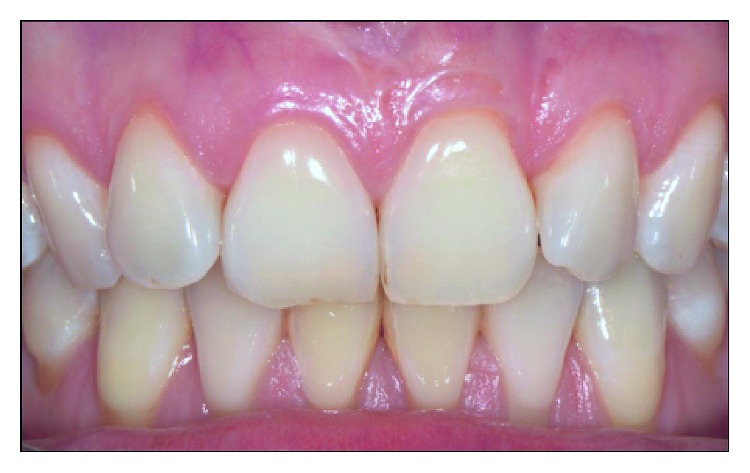
Clinical conditions 15 years after the surgical intervention.

The patient was recalled 3, 6, and 12 months after surgery and annually after. Professional oral hygiene was performed in each follow-up visit up to 15 years. After one year, a significant reduction of the gingival recession was observed with an almost complete root coverage (Figure 7). This clinical condition remained stable also after 15 years (Figure 8). The PES was 8, and WES was 8 highlighting an improvement also in aesthetic appearance.

## 3. Discussion

The case report described above shows the long-term follow-up of one case of surgical retreatment of one gingival recession in the aesthetic area in a young patient.

As a result of the surgical retreatment, the aspect of the gingival tissues improved, with a complete root coverage and the removal of the scars from the failure of a prior surgical intervention. Fifteen years after the surgical intervention, the result remained unchanged.

As it was observed, scars occurred less frequently at the level of the oral mucosa than skin, probably due to the faster healing of the epithelial tissues of the oral cavity or to the presence of saliva [24]. The presence or persistence of inflammation during the healing process could be recognized as one factor related to scar formation [24, 25].

Some authors have related the formation of scars to a trauma that occurred during surgery to the periosteum that could lead to the formation of fibrous tissues during the healing period [17]. This could occur during surgery when vertical incisions were performed [26]. One study performed investigating scar formation after apical surgery evaluated the outcomes after 1 year of 57 apical surgeries [27]. In the study on scar formation after apical surgery, the outcomes of 57 cases were evaluated after one year follow-up. Authors failed to demonstrate a correlation between some of the tested variables (age, gender, jaw, region, duration of surgery, and time of suture removal) but stressed out the importance of an adequate surgical protocol to avoid the formation of scars [27].

Moreover, the presence of scar tissue could impair importantly the aesthetic outcome of the treatment even in cases when complete root coverage was obtained [28].

To our knowledge, there are no studies (either prospective or retrospective) in the literature on the number of retreatments after failure of the surgical intervention for gingival recession coverage. However, from the technical point of view, the procedure that was performed can be supported by sound scientific evidence.

A few randomized controlled clinical trials compared CAF and CAF + CTG for the treatment of the single gingival recession defects showing short-term (6 months) results [15, 29]. In 2004, Da Silva and coworkers reported that the application of a CTG as an adjunct to CAF could significantly improve the treatment compared to CAF alone [29]. More recently, the results of a multicentric randomized controlled clinical trial were published by Cortellini and coworkers in 2009 [15]. The authors reported better clinical results of CAF + CTG compared to CAF.

Even though some authors found that short-term results after CAF procedures could be a reliable predictor of the medium-term results (3 years), just few studies presented long-term results.

One randomized clinical trial published in 2013 presented the 5-year clinical results of CAF or CAF + CTG for the treatment of single gingival recession [30]. Considering the 5-year residual recession, the CAF + CTG group showed superior results (0.19 ± 0.44 mm) than the CAF group (0.46 ± 0.60 mm). Furthermore, 5 years after surgery, in the CAF + CTG group, 82.5% of sites presented complete root coverage (CRC), while 59.6% of sites treated with CAF alone showed CRC. The authors concluded that CAF + CTG could be considered more effective than CAF alone for the treatment of single gingival recession.

As for the choice of the treatment approach, the existing literature provided a valid support for using CAF + CTG technique in the presented case, hence allowing to augment the width of the gingival tissue to correct the aesthetic appearance.

Even though in the present case report the substantial stability of the gingival tissue level was evaluated, it cannot be predicted how it will evolve over the time in a longer period.

Considering the long-term outcomes of the described case report, it can be affirmed that the treatment of the single gingival recession can be successfully performed, even in the presence of scar formation, using the microsurgical technique, and stable results could be maintained long term.

Further research performed on the large sample could help in better understanding of the efficacy of surgical retreatment of gingival recessions.
